# Taking the lid off ambitions for optical imaging of the human brain: a conversation with Clare Elwell

**DOI:** 10.1117/1.NPh.10.2.020401

**Published:** 2023-03-20

**Authors:** Ilias Tachtsidis

**Affiliations:** University College London, London, United Kingdom

## Abstract

Ilias Tachtsidis, professor of biomedical engineering, senior member of the Biomedical Optics Research Laboratory, and head of the Multi-Modal Spectroscopy Group at University College London (UCL), interviewed his colleague and mentor Clare Elwell, professor of medical physics at UCL and Vice Dean of Impact for UCL Engineering, about her pioneering work in fNIRS and brain imaging for global health.

**Figure:**
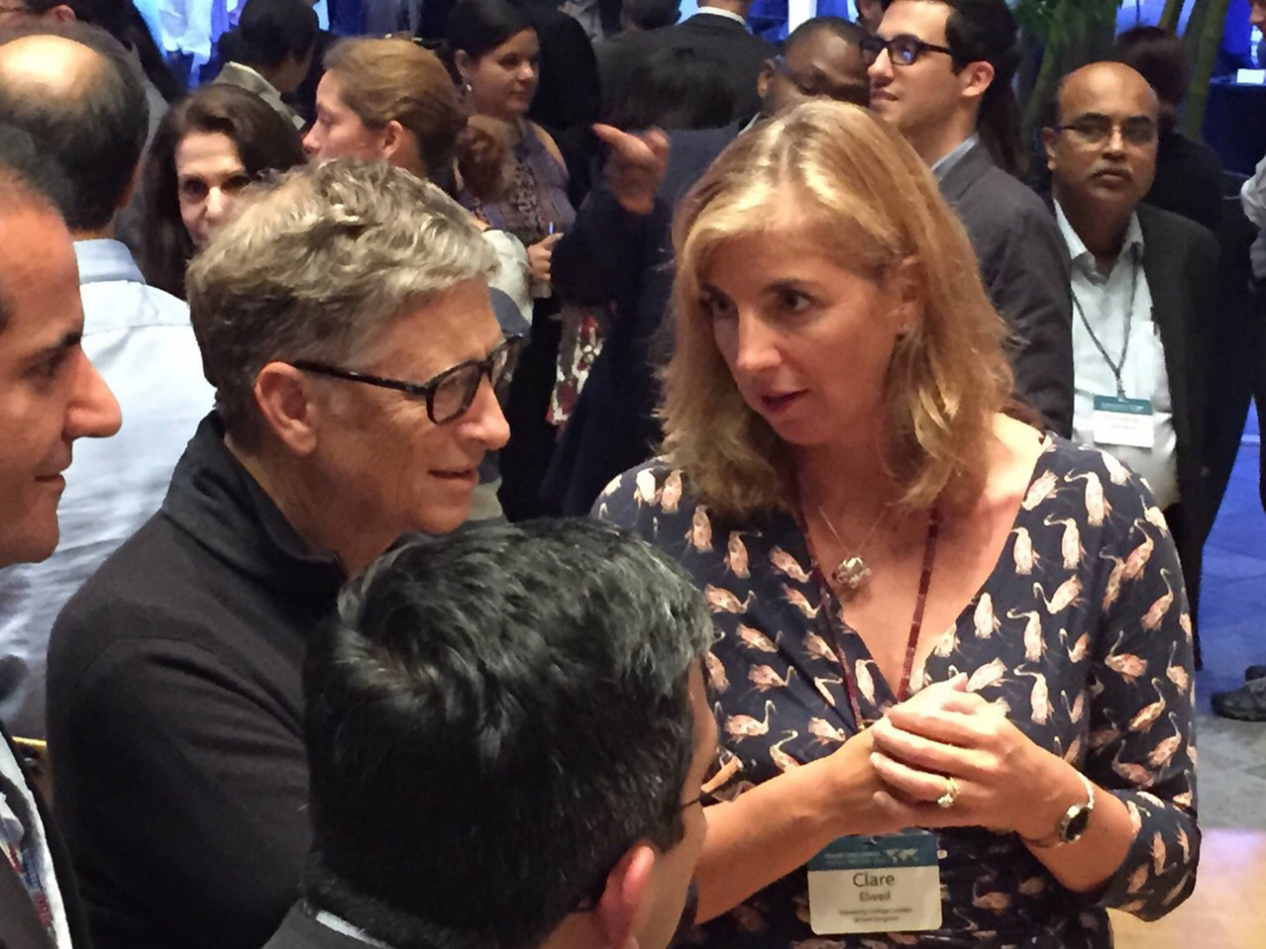
Bill Gates meets Clare Elwell (2014): in conversation with her colleague Ilias Tachtsidis, Clare Elwell reflects on the communication and teamwork involved in the Brain Imaging for Global Health (BRIGHT) project, among other topics. View the video at https://doi.org/10.1117/1.NPh.10.2.020401

